# Gene-Targeted Mice with the Human Troponin T R141W Mutation Develop Dilated Cardiomyopathy with Calcium Desensitization

**DOI:** 10.1371/journal.pone.0167681

**Published:** 2016-12-09

**Authors:** Mohun Ramratnam, Guy Salama, Ravi K. Sharma, David Wen Rui Wang, Stephen H. Smith, Sanjay K. Banerjee, Xueyin N. Huang, Lindsey M. Gifford, Michele L. Pruce, Bethann E. Gabris, Samir Saba, Sanjeev G. Shroff, Ferhaan Ahmad

**Affiliations:** 1 Division of Cardiovascular Medicine, Department of Medicine, University of Wisconsin, Madison, WI, United States of America; 2 Cardiology Section, Medical Service, William. S. Middleton Memorial Veterans Hospital, Madison, WI, United States of America; 3 UPMC Heart and Vascular Institute and Division of Cardiology, Department of Medicine, University of Pittsburgh, Pittsburgh, PA, United States of America; 4 Department of Bioengineering, University of Pittsburgh, Pittsburgh, PA, United States of America; 5 Division of Cardiovascular Medicine, Department of Internal Medicine, University of Iowa Carver College of Medicine, Iowa City, IA, United States of America; 6 Abboud Cardiovascular Research Center, University of Iowa, Iowa City, IA, United States of America; Tokyo Ika Shika Daigaku, JAPAN

## Abstract

Most studies of the mechanisms leading to hereditary dilated cardiomyopathy (DCM) have been performed in reconstituted *in vitro* systems. Genetically engineered murine models offer the opportunity to dissect these mechanisms *in vivo*. We generated a gene-targeted knock-in murine model of the autosomal dominant Arg141Trp (R141W) mutation in *Tnnt2*, which was first described in a human family with DCM. Mice heterozygous for the mutation (*Tnnt2*^R141W/+^) recapitulated the human phenotype, developing left ventricular dilation and reduced contractility. There was a gene dosage effect, so that the phenotype in *Tnnt2*^R141W/+^mice was attenuated by transgenic overexpression of wildtype *Tnnt2* mRNA transcript. Male mice exhibited poorer survival than females. Biomechanical studies on skinned fibers from *Tnnt2*^R141W/+^ hearts showed a significant decrease in pCa_50_ (-log[Ca^2+^] required for generation of 50% of maximal force) relative to wildtype hearts, indicating Ca^2+^ desensitization. Optical mapping studies of Langendorff-perfused *Tnnt2*^R141W/+^ hearts showed marked increases in diastolic and peak systolic intracellular Ca^2+^ ([Ca^2+^]_i_), and prolonged systolic rise and diastolic fall of [Ca^2+^]_i_. Perfused *Tnnt2*^R141W/+^ hearts had slower intrinsic rates in sinus rhythm and reduced peak heart rates in response to isoproterenol. *Tnnt2*^R141W/+^ hearts exhibited a reduction in phosphorylated phospholamban relative to wildtype mice. However, crossing *Tnnt2*^R141W/+^ mice with phospholamban knockout (*Pln*^-/-^) mice, which exhibit increased Ca^2+^ transients and contractility, had no effect on the DCM phenotype. We conclude that the *Tnnt2* R141W mutation causes a Ca^2+^ desensitization and mice adapt by increasing Ca^2+^-transient amplitudes, which impairs Ca^2+^ handling dynamics, metabolism and responses to β-adrenergic activation.

## Introduction

Familial cardiomyopathies are primary disorders of the myocardium caused by heritable mutations in single genes, the most common of which are hypertrophic (HCM) and dilated cardiomyopathy (DCM) [1, 2]. DCM is characterized by left ventricular or biventricular dilation and depressed myocardial contractility. Its incidence is 8/100,000, and its prevalence 36/100,000. Once patients are symptomatic, mortality is 25% at one year and 50% at 5 years [3]. Considerable progress has been made in the past two decades in determining the molecular genetic basis for DCM and HCM, with mutations in genes encoding sarcomere proteins accounting for a significant fraction of DCM and a majority of HCM cases. A particularly intriguing discovery has been that mutations in some of the same genes, such as the gene encoding cardiac troponin T (cTnT, *TNNT2*) can lead to either DCM or HCM [4]. cTnT is a component of the troponin complex within the thin filament of the sarcomere which allows actomyosin interaction and contraction to occur in response to Ca^2+^.

Despite the identification of causative genetic mutations, the mechanisms leading to the phenotype are incompletely understood. For DCM mutations, Ca^2+^ desensitization and/or decreased ATPase activity have been generally observed [5]. We [4] and others [6, 7] have shown using *in vivo* and *in vitro* approaches that Ca^2+^ desensitization plays a major role in the pathophysiology of the DCM *TNNT2* K210Δ mutation. In addition, while Ca^2+^ desensitization has been observed *in vitro* in the DCM *TNNT2* Arg141Trp (R141W) mutation [8–11], there have also been some discrepant results such as a failure to observe an effect of the R141W mutation on Ca^2+^ sensitivity [12]. In contrast, most studies have demonstrated an increase in Ca^2+^ sensitivity resulting in increases in force generation, ATPase activity, and hypercontractility in HCM mutations [5].

The majority of these studies were performed in reconstituted *in vitro* systems. Genetically engineered murine models offer the opportunity to dissect the mechanisms leading from genotype to phenotype *in vivo*. We [4] and others have generated a few transgenic murine models of human mutations leading to DCM. However, transgenic models that express mutant cTnT or “poison peptide” do not completely duplicate phenotypes typical of human DCM. The degree of overexpression of the transgene cannot be controlled to reflect levels observed in human patients, a critical consideration because different concentrations of mutant cTnT may produce varying effects [13]. Gene targeting better reflects human mutations by replacing an endogenous gene with a mutant allele at a defined site, leaving it under the physiological genomic control. Previously, we [4] reported on a transgenic murine model and others [7] reported on a gene-targeted knock-in murine model of DCM caused by the *Tnnt2* K210Δ mutation, both of which exhibited Ca^2+^ desensitization. Here, we have generated a gene-targeted knock-in murine model of the autosomal dominant Arg141Trp (R141W) mutation in *Tnnt2*, which was first described in a human family with DCM [14]. In this study, we established the effect of gene dosage in phenotype severity, uncovered sex dependent differences in survival, and identified molecular and biomechanical mechanisms of ventricular remodeling. In addition, since the mutation is expected to decrease Ca^2+^ sensitivity, we investigated its effects on Ca^2+^ homeostasis using molecular, biochemical, and optical mapping techniques.

## Methods

### Genetically engineered animal models

**Fig 1:**
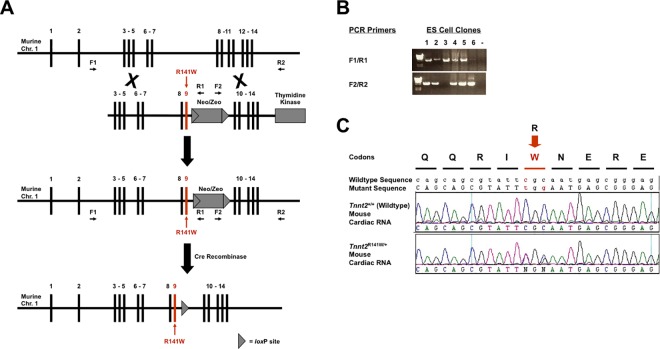
Generation of *Tnnt2*^R141W/+^ mice. **(A)** Homologous recombination strategy used to introduce the DCM Arg141Trp (R141W) mutation into exon 9 of the murine *Tnnt2* gene in ES cells. The resultant mice were mated with EIIa-Cre recombinase mice to remove the floxed *neo/zeo* cassette. **(B)** ES clones having undergone successful homologous recombination with the R141W targeting construct were identified by long range PCR using primer pairs F1 and R1 at the 5’ end, and F2 and R2 at the 3’ end. Positions of the primers are shown in panel A. Clones 1, 2, 4, and 5 showed appropriate homologous recombination at both the 5’ and the 3’ ends. **(C)** Expression of mutant *Tnnt2* mRNA in *Tnnt2*^R141W/+^ mice was confirmed by reverse transcription-PCR and sequencing of total cardiac RNA. WT, wildtype.

Fig 1A illustrates the strategy to introduce the human DCM *TNNT2* Arg141Trp mutation (R141W) [14] into the mouse genome by homologous recombination in murine 129/SvEv derived ES cells [15]. A murine genomic segment containing the *Tnnt2* gene was subcloned from the CitbCJ7 BAC library, clone 353I2 (Invitrogen), into a plasmid containing a *neo/zeo* resistance cassette, and a thymidine kinase gene. The mutation in exon 9 was generated by PCR. The construct was linearized and electroporated into 129/SvEv strain TC1 ES cells (a generous gift from Philip Leder, M.D., Harvard Medical School, Boston, MA). Targeted ES cells were selected with Genetecin/G418 (Invitrogen) and FIAU (Moravek), and incorporation of the construct by homologous recombination detected by long range PCR using primers within the construct and in the murine genome adjacent to the construct (Fig 1B). These ES cells were used to generate chimeras by blastocyst injection, which were then bred to generate heterozygous mice with one mutant *Tnnt2* allele. The *neo/zeo* cassette was excised by mating these mice with 129/SvEv strain EIIa-Cre recombinase transgenic mice [16], to generate progeny with the R141W mutation in the endogenous murine *Tnnt2* gene, designated *Tnnt2* ^R141W/+^. Expression of the mutant *Tnnt2* allele was confirmed by reverse transcription-PCR and sequencing of the *Tnnt2* transcript from total cardiac mRNA (Fig 1C). Heterozygous *Tnnt2* ^R141W/+^ mice were crossbred to generate homozygous *Tnnt2* ^R141W/R141W^ mice.

We have previously reported on our transgenic mice overexpressing wildtype cTnT (TG^WT^) [4]. These mice were backcrossed for at least 10 generations from an FVB to a 129/SvEv background, and then crossed with *Tnnt2* ^R141W/R141W^ mice to generate *Tnnt2*^R141W/+^/TG^WT^ mice. Phospholamban knockout mice (*Pln*^-/-^) were a generous gift from Evangelia Kranias, Ph.D., University of Cincinnati [17]. These mice were backcrossed for at least 10 generations to a 129/SvEv background, and then crossed with *Tnnt2* ^R141W/R141W^ mice to generate *Tnnt2* ^R141W/+^/*Pln*^-/-^ mice.

For the assessment of long-term survival, mice were monitored daily for signs of heart failure, including tachypnea, dyspnea, lethargy, weight loss, piloerection, changes in posture to ease breathing discomfort (supporting head up on objects in cage), or a ‘bulging’ appearance of the eyes. Mice that exhibited these signs were immediately euthanized rather than being palliated with analgesics, and their hearts excised to confirm dilation. Some mice exhibited sudden cardiac death without any prior signs of heart failure, and necropsies failed to identify non-cardiac causes of death.

All studies conformed to the *Guide for the Care and Use of Laboratory Animals*, published by the National Institutes of Health, and were approved by the University of Pittsburgh and the University of Iowa Institutional Animal Care and Use Committees (IACUCs), protocols # 1101022 and 1311199, respectively. For studies requiring sedation, mice were administered tribromoethanol 125–375 mg/kg IP once and the level of sedation was monitored by reduced response to toe or tail pinch. Unless indicated otherwise, for studies requiring euthanasia, mice underwent CO_2_ narcosis by inhalation from a compressed gas cylinder, followed by cervical dislocation, thoracotomy, and harvest of the heart.

### RNA analyses

RNA extraction, reverse transcription PCR extraction, cDNA sequencing, and real-time quantitative PCR (QPCR) were performed as we have previously described [18–20]. Briefly, total RNA was isolated from whole heart with TRIzol (Invitrogen). Reverse transcriptase reactions were performed using the Superscript III First-Strand Kit (Invitrogen) for first-strand cDNA synthesis. Primers for QPCR analysis for each transcript of interest are listed in Table 1. cDNA (10 ng) was analyzed on an ABI PRISM 7700 using Absolute SYBR Green ROX PCR Master Mix (Thermo Scientific). Fold-changes were calculated after normalization to cyclophilin transcript levels. The annealing temperature was 60°C.

**Table 1:** Primer sequences used for real-time quantitative PCR (QPCR).

Transcript	Direction	Primer Sequence (5' to 3')
Mutant *Tnnt2*	Forward	CGAGCAGCAGCGTATTTGG
	Reverse	CAGCCTTCCTCCTGTTCTCCTC
Wildtype *Tnnt2*	Forward	CGAGCAGCAGCGTATTCGC
	Reverse	CAGCCTTCCTCCTGTTCTCCTC
Total *Tnnt2*	Forward	GGGCCGAGCAGCAGCGTATT
	Reverse	CAGCCTTCCTCCTGTTCTCCTC

### Immunoblots

Protein analyses were performed as we have previously described [21–23]. 10–20 μg of protein per lane were loaded onto an appropriate concentration protein gel (Pierce), subjected to electrophoresis at 200 V for 45 min, and transferred from the gel to a PVDF membrane. The membrane was blocked and incubated at 4°C overnight with primary antibody at the recommended dilutions, followed by the appropriate horseradish peroxidase-conjugated secondary antibody (Santa Cruz, Amersham, or Cell Signaling) at the appropriate dilution at room temperature for 45–60 min. Proteins were visualized and quantified by enhanced chemiluminescence (Pierce). Signal intensity was normalized to protein loading using GAPDH immunoblots or Coomassie blue gel staining. Prior to immunoblotting for Janus kinase 1 (Jak1), Jak2, and Stat3, samples were immunoprecipitated by incubation overnight with the antibody of interest at 4°C, and then conjugated and pulled down with protein A agarose beads (Pierce). The following primary antibodies were used for immunoblots: from Affinity BioReagents, calsequestrin 2 (CASQ2, #pA-913), sarcoplasmic reticulum type slow twitch skeletal muscle isoform cardiac Ca^2+^ ATPase (SERCA2, #MA3-919); from Cell Signaling, Akt (#9272), phospho-Ser^473^ Akt (#9271), phospho-troponin I (Cardiac) (Ser23/24) (#4004), phospho-Thr^202^/Tyr^204^ extracellular signal-regulated kinase mitogen-activated protein kinase **(**ERK) 1/2 (#9101), p38 mitogen-activated protein kinase (MAPK) (#9212), phospho-Thr^180^/Tyr^182^ p38 mitogen-activated protein kinase (MAPK) (#9211S), Jak1 (#3344), phospho-Jak1 (#3331), Jak2 XP (#3230), phospho-Jak2 (#3771), Stat3 (#4904), phospho-Stat3 (#9131); from Fitzgerald, GAPDH (#RDI-TRK5G4-6C5); from Santa Cruz, troponin I (H-170) (sc-15368), cardiotrophin-1 (#sc-20867), sodium/calcium exchanger (NCX1) (#sc-30306); from Upstate, phospholamban (PLN, #05–205), phosphor-Ser^16^-phosphlamban (PLN, #07–052),.

### Echocardiography

Transthoracic echocardiography was performed on a VisualSonics Vevo 770 machine [4]. Mice were sedated with tribromoethanol (125 mg/kg IP). Left ventricle end diastolic (LVEDD) and end systolic (LVESD) chamber dimensions and wall thickness (LVWT) were obtained from M-mode tracings based on measurements averaged from three separate cardiac cycles. LV fractional shortening (FS) was calculated as (LVEDD—LVESD) / LVEDD x 100%.

### Biomechanical studies

Biomechanical studies were performed as we have described [4]. Briefly, anterior LV papillary muscles were cut into strips and skinned overnight in relaxing solution (in mM, 40 BES [pH 7.0], 20 KCl, 1 free Mg^2+^, 5 MgATP, 10 creatine phosphate, 20 EGTA, 1 DTT, 0.01 leupeptin, 0.1 PMSF, 180 ionic strength) with 1% Triton X-100 at 4°C. Skinned muscle bundles were mounted in a 750 μl bath and were attached to a length controller (model 322B, Aurora Scientific) at one end and a force transducer (model 403A, Aurora Scientific) at the other end. Sarcomere length (SL) was determined using laser diffraction (Spectra-Physics, 10 mW HeNe laser) and set at either 1.9 or 2.3 μm. Activating solution (pCa 4.33) contained all the ingredients of relaxing solution (pCa 10). Force-pCa data were collected by exposing the skinned fiber to various concentrations of free Ca^2+^ (pCa range: 7.00–4.33) that were generated by mixing relaxing and activating solutions in appropriate proportions.

Normalized force was calculated as the ratio of the measured force at a given pCa and the maximally activated force (i.e., force at pCa = 4.33). Normalized force-pCa data were fitted to a modified Hill equation [24] using a nonlinear regression algorithm (Prism, GraphPad Software). Two parameters were estimated from normalized force-pCa data for each fiber: pCa_50_ (-log[Ca^2+^] required to produce normalized force of 50%) and Hill coefficient (a measure of the steepness of the normalized force-pCa curve, which characterizes the cooperative phenomena in muscle force generation).

### Electrocardiography (ECG) and electrophysiological studies

An octapolar catheter (NuMed) was advanced through the right external jugular vein to the right ventricular apex. Programmed ventricular stimulation, consisting of burst pacing at cycle lengths of 100 ms to 50 ms in decrements of 10 ms, and of programmed stimulation with double and triple extrastimuli at a drive cycle length of 100 ms with a coupling interval ≥ 30 ms, were performed. Inducibility was defined as 10 beats of ventricular tachycardia (VT) [4].

### Optical maps of action potentials and intracellular Ca^2+^ transients

Mice were anaesthetized with pentobarbital (50 mg/kg) and heparinized (35 mg/kg) intraperitoneally. The heart was rapidly excised, cannulated and perfused (2.5 mL/min) with standard oxygenated Tyrode’s solution. The heart was oriented such that the anterior surface of the ventricles was imaged on a photodiode array (16x16 element Hammamatsu, Hammamatsu City, Japan; no. C4675-103). The cardiac perfusate contained (in mM) 112 NaCl, 1.0 KH_2_PO_4_, 25.0 NaHCO_3,_ 1.2 MgSO_4_, 5.0 KCl, 50.0 dextrose, 1.8 CaCl_2_, at pH 7.4, and was gassed with 95% O_2_ and 5% CO_2_. Perfusion pressure was adjusted to 60–80 mmHg by controlling the flow rate of a peristaltic pump. The temperature of the bath surrounding the heart was kept at 37°C. All recordings were made in the absence of chemical uncouplers such as diacetyl monoxime or cytochalasin D. Motion artifacts were suppressed by mechanical stabilization with the chamber and extensive controls were carried out to ensure that immobilization of the heart did not make the heart ischemic, as previously described [25–27].

Hearts were stained with a voltage-sensitive dye, di-4-ANEPPS by injecting 40 μl of stock solution (1 mg dye per ml DMSO) to map action potential (AP) propagation [25] or with a Ca^2+^ indicator Rhod-2/AM (300 μl of 1 mg/ml DMSO) to measure Ca^2+^ transients [28]. The single excitation-emission Ca^2+^ indicator Rhod-2 was used because it is localized in the cytosol (if loaded at 37°C for 5 min), has low toxicity, and is stable for several hours for long-lasting intracellular Ca^2+^ recordings [26, 29]. In addition, Rhod-2 signals can be calibrated to measure the absolute concentration of free cytosolic Ca^2+^. Ca^2+^ calibrations were necessarily performed in the absence of a voltage sensitive dye and were accomplished by measuring F_max_ (maximum Rhod-2 fluorescence when saturated with Ca^2+^) and F_min_ (minimum Rhod-2 fluorescence when all Ca^2+^ is washed out and chelated with EGTA), as previously described [30]. Although maps of optical action potentials (APs) shown here were recorded with di-4-ANEPPS, in the absence of a Ca^2+^ indicator, simultaneous optical mapping of APs and Ca^2+^-transients using RH 237 and Rhod-2 showed normal voltage-Ca^2+^ delays in *Tnnt2* ^R141W/+^ compared to control littermate mice (not shown). Bipolar Ag^+^-AgCl electrodes (250 μm diameter Teflon-coated silver wire) were placed on the apical and basal surfaces of the ventricle and used to stimulate or record electrograms in parallel with the 256 optical action potentials [25, 30].

### Data analysis

In general, quantitative data are presented in tables and graphs as mean ± standard deviation (SD), unless otherwise indicated. Comparisons between two groups were made using the Student’s *t*-test. For comparisons among three or more groups, we used one-way ANOVA followed by *post hoc* Bonferroni correction for echocardiography data and the Tukey’s test for densitometry analysis. Force-pCa data were analyzed using two-way (mouse type and sarcomere length) ANOVA with one repeated measure (sarcomere length), with *post hoc* comparisons using the Tukey’s test. Differences in inducibility of arrhythmias were analyzed by the chi-squared test. Survival differences were analyzed by the log-rank test.

## Results

### Expression of mutant *Tnnt2* R141W mRNA transcript in *Tnnt2*^R141W/+^ mice

Gene-targeted (knock-in) mice with a mutation in *Tnnt2* previously discovered in human families with DCM [14] were generated by homologous recombination (Fig 1A). Expression of the mutant allele on one chromosome and the endogenous wildtype allele on the other was confirmed by reverse transcription-PCR of total cardiac RNA with *Tnnt2*-specific primers and direct sequencing (Fig 1C). The presence of two peaks on the electrophoregram representing codons CGC (encoding the wildtype Arg residue) and TGG (encoding the mutant Trp residue) indicates that both alleles were expressed in heterozygous mice, analogous to human patients with this mutation. Using primers specific for total *Tnnt2* transcript, wildtype *Tnnt2* transcript, and mutant *Tnnt2* transcript, we conducted QPCR to determine the relative expression of wildtype and mutant *Tnnt2* transcript in cardiac tissue from mice of each genotype. Mutant *Tnnt2* transcript was undetec in wildtype mice. In contrast, the ratios of wildtype to total *Tnnt2* transcript and mutant to total *Tnnt2* transcript in *Tnnt2*^R141W/+^ mice were each approximately 50% (*P<*0.001, Table 2). It should be noted that the relative expression of wildtype and mutant cTnT protein may not be exactly the same as the relative expression of wildtype and mutant *Tnnt2* mRNA transcript.

**Table 2:** Relative cardiac expression of wildtype and mutant *Tnnt2* mRNA.

	Wildtype / Total *Tnnt2* mRNA	Mutant / Total *Tnnt2* mRNA
*Tnnt2*^+/+^ (WT)	1.00 ± 0.04	0.00 ± 0.00 ^†^
TG^WT^	1.24 ± 0.09	0.00 ± 0.00 ^†^
*Pln*^-/-^	1.16 ± 0.15	0.00 ± 0.00 ^†^
*Tnnt2*^R141W/+^	0.55 ± 0.03 *	0.62 ± 0.03 *
*Tnnt2*^R141W/+^/TG^WT^	1.17 ± 0.05 ^†^	0.17 ± 0.01 *^,^^†^
*Tnnt2*^R141W/+^/*Pln*^-/-^	0.44 ± 0.07 *	0.49 ± 0.03 *
*Tnnt2*^R141W/R141W^	0.00 ± 0.00 *^,^^†^	1.00 ± 0.08 *^,^^†^

*n* = 4 / group.

* *P<*0.001 versus wildtype

^†^ *P<*0.001 versus *Tnnt2*^R141W/+^.

### Development of DCM at an early age in *Tnnt2*^R141W/+^ mice

**Fig 2:**
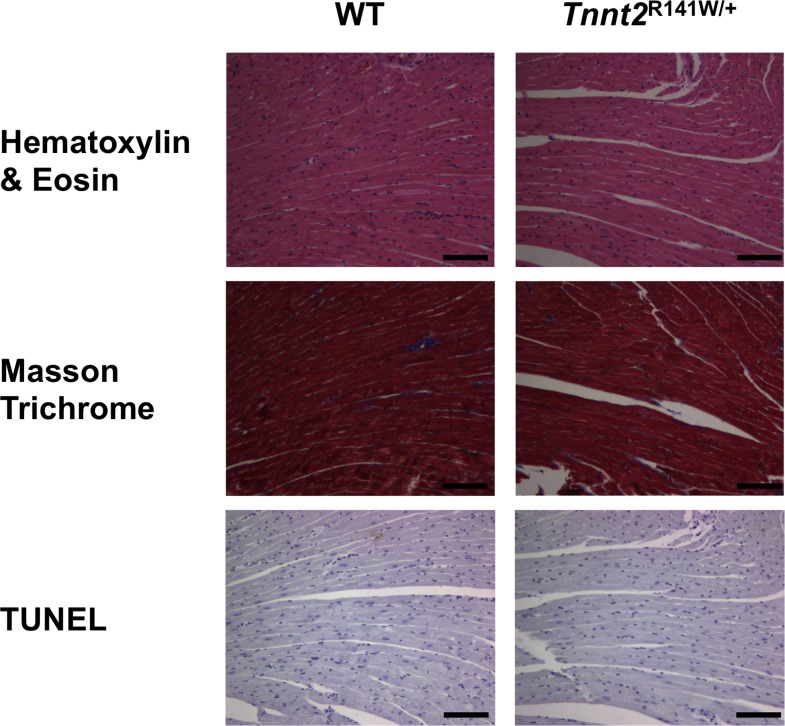
Cardiac histopathology. Cardiac tissue sections from 15-week-old wildtype (WT) and *Tnnt2*^R141W/+^ mice were stained with hematoxylin and eosin, Masson trichrome, and TUNEL. Despite the emergence of DCM, no significant myofibrillar disarray, fibrosis, or apoptosis was evident in *Tnnt2*^R141W/+^ hearts. Scale bar = 100 μm.

Echocardiography showed significant increases in left ventricular end diastolic diameter (LVEDD) and decreases in fractional shortening (FS) in both male and female *Tnnt2*^R141W/+^ mice relative to wildtype mice (Table 3), indicating that these mice recapitulated the human phenotype of LV dilation and reduced systolic function. This DCM phenotype was evident at age 6 weeks, and persisted at ages 12 and 30 weeks. Myofibrillar disarray, although typical of HCM, is observed variably in DCM. Cardiac tissue sections from 16-week-old *Tnnt2*^R141W/+^ mice were stained with hematoxylin and eosin, Masson trichrome, and TUNEL (Fig 2). No significant myofibrillar disarray, fibrosis, or apoptosis was evident in the *Tnnt2*^R141W/+^ mice. On invasive electrophysiological studies, 0 of 12 wildtype mice exhibited inducible ventricular tachyarrhythmias while 2 of 4 *Tnnt2*^R141W/+^ mice exhibited nonsustained polymorphic ventricular tachycardia on aggressive programmed ventricular stimulation (*P<*0.01 for inducibility).

**Table 3:** Cardiac morphology and function of mice assessed by echocardiography.

	*Tnnt2*^+/+^ (WT)	*Tnnt2*^R141W/+^	*Tnnt2*^+/+^ (WT)	*Tnnt2*^R141W/+^	*Tnnt2*^+/+^ (WT)	*Tnnt2*^R141W/+^
Sex	Male and Female	Male and Female	Male	Male	Female	Female
**6 Weeks**						
No. of mice	7	10	4	6	3	4
LVWT (mm)	0.76 ± 0.04	0.61 ± 0.05	0.73 ± 0.06	0.57 ± 0.04 ^†^	0.81 ± 0.05	0.66 ± 0.1
LVEDD (mm)	3.53 ± 0.10	4.08 ± 0.10 *	3.71 ± 0.09	4.11 ± 0.13 ^†^	3.29 ± 0.09 ^§^	4.04 ± 0.17 ^‡^
LVESD (mm)	2.21 ± 0.10	3.14 ± 0.14 *	2.26 ± 0.18	3.08 ± 0.02 ^†^	2.15 ± 0.08	3.22 ± 0.21^‡^
FS (%)	37 ± 3	23 ± 2 *	39 ± 5	25 ± 3 ^†^	34 ± 1	21 ± 2 ^‡^
HR (bpm)	425 ± 17	420 ± 15	416 ± 20	405 ± 22	436 ± 34	441 ± 15
**12 Weeks**						
No. of mice	11	27	6	11	5	16
LVWT (mm)	0.82 ± 0.03	0.67 ± 0.09 *	0.81 ± 0.03	0.67 ± 0.03 ^†^	0.84 ± 0.07	0.67 ± 0.03 ^‡^
LVEDD (mm)	3.39 ± 0.10	3.93 ± 0.08 *	3.64 ± 0.06	4.17 ± 0.07 ^†^	3.09 ± 0.06 ^§^	3.76 ± 0.11 ^‡^^||^
LVESD (mm)	2.22 ± 0.07	2.96 ± 0.09 *	2.38 ± 0.05	3.2 ± 0.12 ^†^	2.02± 0.07 ^§^	2.79 ± 0.1 ^‡^^||^
FS (%)	35 ± 2	25 ± 1 *	34 ± 2	23 ± 2 ^†^	35 ± 3	26 ± 2 ^‡^
HR (bpm)	426 ± 18	430 ± 10	457 ± 23	439 ± 16	389± 18	423 ± 13
**30 Weeks**						
No. of mice	14	8	8	4	6	4
LVWT (mm)	0.77 ± 0.04	0.64 ± 0.03	0.8 ± 0.06	0.62 ± 0.02 ^†^	0.73 ± 0.05	0.66 ± 0.07
LVEDD (mm)	3.64 ± 0.11	3.88 ± 0.11 *	3.87 ± 0.12	4.03 ± 0.14	3.34 ± 0.11 ^§^	3.64 ± 0.19
LVESD (mm)	2.37 ± 0.09	2.93 ± 0.12 *	2.46 ± 0.11	3.11 ± 0.15 ^†^	2.25 ± 0.16	2.75 ± 0.14 ^‡^
FS (%)	35 ± 2	22 ± 1 *	37 ± 2	23 ± 1 ^†^	33 ± 3	25 ± 2 ^‡^
HR (bpm)	421 ± 14	428 ± 15	424 ± 21	419 ± 25	417 ± 22	449 ± 26

*, *P*<0.05 Male and Female WT vs Male and Female *Tnnt2*^R141W/+^

^†^, *P*<0.05 Male WT vs Male *Tnnt2*^R141W/+^

^‡^, *P*<0.05 Female WT vs. Female *Tnnt2*^R141W/+^

^§^, *P*<0.05 Male WT vs. Female WT

^||^, *P*<0.05 Male *Tnnt2*^R141W/+^ vs. Female *Tnnt2*^R141W/+^

### Sex-related differences in survival

**Fig 3:**
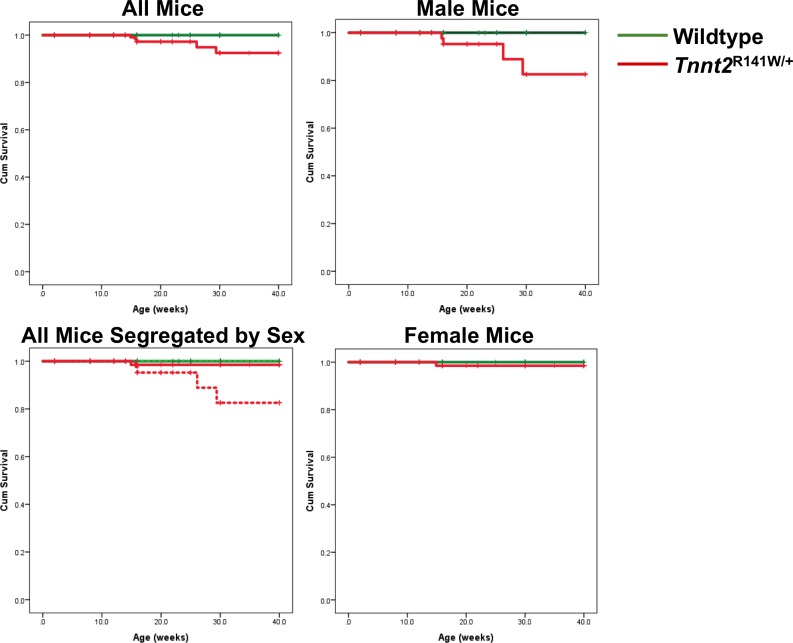
Kaplan-Meier survival curves. Data are presented for all genotypes for all mice (upper left), all mice segregated by sex (lower left), all male mice (upper right), and all female mice (lower right). In the lower left panel, female mice are designated by solid lines, and male mice by broken lines. *P*<0.001 for all comparisons except *Tnnt2*^R141W/+^ females versus wildtype females and wildtype males versus wildtype females. *n* = 84 wildtype males, 67 wildtype females, 79 *Tnnt2*^R141W/+^ males, 100 *Tnnt2*^R141W/+^ females.

Not surprisingly, *Tnnt2*^R141W/+^ mice exhibited poorer survival than wildtype mice (Fig 3). However, female *Tnnt2*^R141W/+^ mice exhibited superior survival rates than male *Tnnt2*^R141W/+^ mice, that were not significantly different from those observed in wildtype mice. Therefore, there may be other genetic and/or endogenous environmental (such as hormonal) modifiers that protect female *Tnnt2*^R141W/+^ mice from DCM-associated mortality. However, these modifiers are currently unknown.

### Attenuation of DCM phenotype by transgenic overexpression of wildtype *Tnnt2* mRNA transcript

We have previously reported on our transgenic mice overexpressing wildtype *Tnnt2* transcript (TG^WT^) [4]. Crossing these mice generated *Tnnt2*^R141W/+^/TG^WT^ mice, which had a significantly reduced ratio of mutant to total *Tnnt2* transcript relative to *Tnnt2*^R141W/+^ mice (Table 1). The transgenic overexpression of wildtype *Tnnt2* transcript, superimposed on expression of R141W mutant *Tnnt2* transcript, largely abrogated the DCM phenotype (Table 4). At age 10 weeks, *Tnnt2*^R141W/+^/TG^WT^ mice had a decreased LVEDD and an improved FS relative to *Tnnt2*^R141W/+^ mice. In fact, there was no significant difference in FS in *Tnnt2*^R141W/+^/TG^WT^ mice relative to WT mice. These data support the presence of a gene dosage effect, such that an increase in the relative expression of wildtype *Tnnt2* transcript attenuated the effect of mutant *Tnnt2* transcript. It should be noted that the relative expression of wildtype and mutant cTnT protein may not be exactly the same as the relative expression of wildtype and mutant *Tnnt2* mRNA transcript shown in Table 2.

**Table 4:** Echocardiography at age 10 weeks in mice crossed with transgenic overexpressors of *Tnnt2* mRNA (TG^WT^) or phospholamban knockout (*Pln*^-/-^) mice.

	No. of mice	LVWT (mm)	LVEDD (mm)	LVESD (mm)	FS (%)	HR (bpm)
*Tnnt2*^+/+^ (WT)	35	0.77 ± 0.02	3.54 ± 0.06	2.33 ± 0.06	34 ± 1	424 ± 9
TG^WT^	7	0.74 ± 0.03	3.53 ± 0.12	2.16 ± 0.12	39 ± 2	398 ± 18
*Pln*^-/-^	8	0.93 ± 0.06	2.92 ± 0.12	1.31 ± 0.13	56 ± 3	532 ± 16
*Tnnt2*^R141W/+^	51	0.66 ± 0.02 *	3.92 ± 0.06 *	3.00 ± 0.07 *	24 ± 1 *	428 ± 7
*Tnnt2*^R141W/+^/TG^WT^	4	0.67 ± 0.09	3.18 ± 0.26 *^,^^**†**^	2.21 ± 0.40 ^**†**^	32 ± 8 ^**†**^	378 ± 14
*Tnnt2*^R141W/+^/*Pln*^-/-^	18	0.75 ± 0.03	3.94 ± 0.12	2.98 ± 0.15 *	25 ± 2 *	486 ± 10

LVWT, left ventricular wall thickness at end diastole; LVEDD, left ventricular end diastolic diameter; FS, fractional shortening; HR, heart rate; bpm, beats per minute; WT, wildtype. *P<*0.01 by ANOVA and Bonferroni correction versus

*WT

^†^*Tnnt2*^R141W/+^.

### Molecular mediators of cardiac remodeling

**Fig 4:**
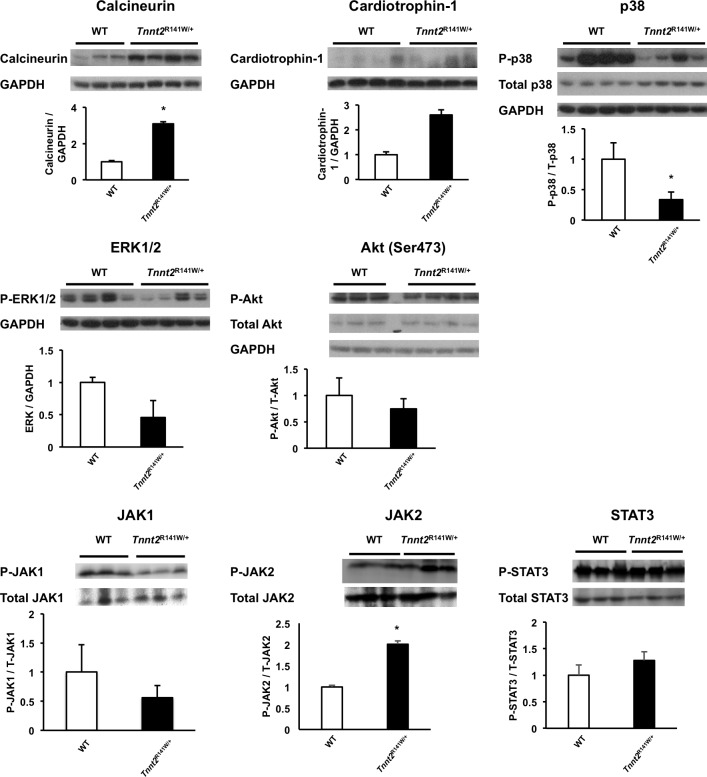
Expression of heart failure signaling pathway proteins. Immunoblots and densitometry analysis are presented of total cardiac protein extracts of heart failure signaling pathways from 2-week-old wildtype (WT) and *Tnnt2*^R141W/+^ hearts. *, *P*<0.05 versus wildtype. *n* = 3–4 for wildtype and *n* = 4 for *Tnnt2*^R141W/+^.

There have been few studies dissecting the molecular mechanisms of cardiac remodeling in gene-targeted murine models of human DCM. We ascertained changes in several signaling pathways at age 2 weeks (Fig 4). The protein expression of calcineurin, a Ca^2+^-regulated phosphatase associated with heart failure and left ventricular hypertrophy, was significantly elevated by 3.1 ± 0.12 fold over wildtype hearts in *Tnnt2*^R141W/+^ hearts (*P<*0.01). Cardiotrophin-1, a member of the interleukin-6 cytokine family that signals through the cytokine receptor gp130, is associated with left ventricular hypertrophy and failure [31]. Immunoblots showed that cardiac expression of cardiotrophin-1 was elevated in *Tnnt2*^R141W/+^ hearts by 2.6 ± 0.2 fold compared to wildtype hearts, although this change did not meet statistical significance (*P* = 0.069). JAK1, JAK2, and STAT3 are activated by cardiotrphin-1. Phosphorylation of JAK2 was significantly increased by 2.0 ± 0.6 fold in *Tnnt2*^R141W/+^ mice compared to wildtype mice (*P*<0.05). The Thr^180^/Tyr^182^ phosphorylated, active form of p38 mitogen-activated protein kinase (MAPK) was significantly downregulated to 0.33 ± 0.13 fold in *Tnnt2*^R141W/+^ hearts relative to wildtype hearts (*P<*0.05). The expression of extracellular signal regulated kinase 1 (ERK1) and ERK2 MAPKs and the Ser^473^ phosphorylated, active form of Akt were not significantly different in *Tnnt2*^R141W/+^ hearts compared to wildtype hearts.

### Myofilament Ca^2+^ desensitization and troponin I (TnI) phosphorylation *in vivo*

**Fig 5:**
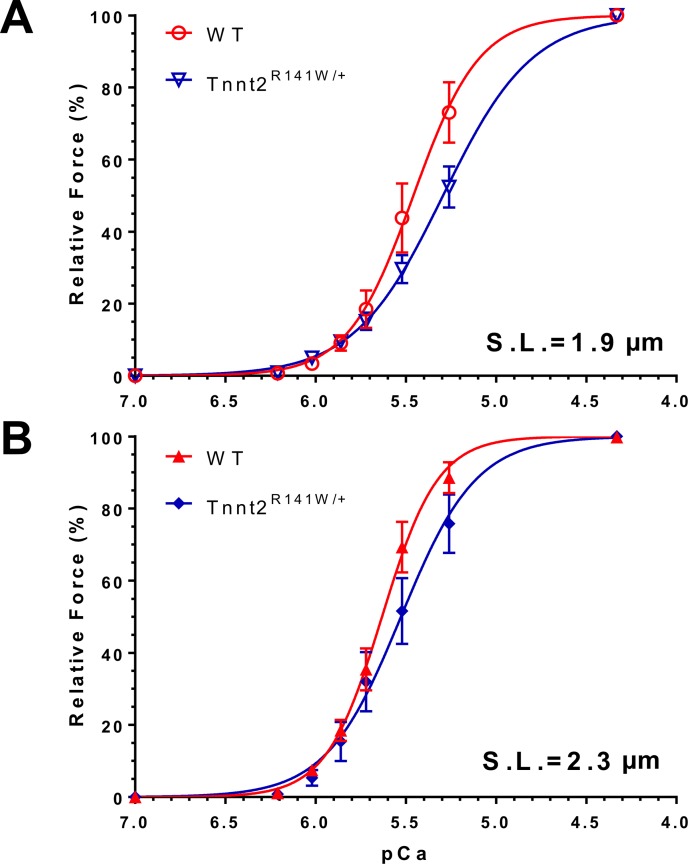
Normalized force-pCa relationships in wildtype and *Tnnt2*^R141W/+^ skinned papillary muscle fibers. Normalized force (i.e., ratio of force at a given pCa and maximally activated force at pCa = 4.33) developed at a range of Ca^2+^ concentrations was assessed at sarcomere lengths 1.9 μm (A: wildtype ○, *Tnnt2*^R141W/+^ ∇) and 2.3 μm (B: wildtype ▲, *Tnnt2*^R141W/+^ ◆). There was a rightward shift of the force-pCa curve in *Tnnt2*^R141W/+^ muscle, indicating Ca^2+^ desensitization. Values are mean±SE (*n* = 8 for wildtype and *n* = 3 for *Tnnt2*^R141W/+^).

**Fig 6:**
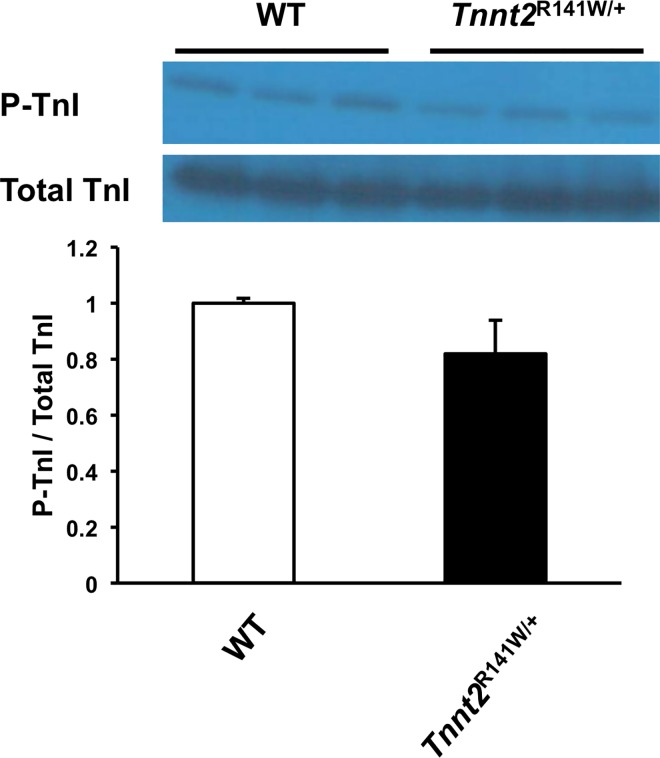
Troponin I expression. Immunoblot and densitometry of phosphorylated troponin I (P-TnI) and total troponin I (TnI) in 8-week-old wildtype (WT) and *Tnnt2*^R141W/+^ hearts. *n* = 3 for wildtype and *n* = 3 for *Tnnt2*^R141W/+^.

Biomechanical studies on skinned fibers from *Tnnt2*^R141W/+^ hearts showed a significantly decreased pCa_50_ at both sarcomere lengths 1.9 and 2.3 μm relative to wildtype hearts, indicating Ca^2+^ desensitization (Table 5 and Fig 5). The Hill coefficient was significantly decreased in *Tnnt2*^R141W/+^ hearts, implying decreased cooperativity. The maximally activated force generation was unchanged. The ratio of phosphorylated to total TnI was unchanged in *Tnnt2*^R141W/+^ hearts compared to wildtype hearts (Fig 6). Therefore, TnI phosphorylation was not responsible for the observed Ca^+^ desensitization.

**Table 5:** Ca^2+^ sensitivity and maximum force generation in skinned papillary muscle fibers.

	*n*	pCa_50_	Hill Coefficient	Maximal Force (mN mm^-2^)	pCa_50_	Hill Coefficient	Maximal Force (mN mm^-2^)
Sarcomere length (μm)		1.9	1.9	1.9	2.3	2.3	2.3
*Tnnt2*^R141W/+^	3	5.41±0.03	2.88±0.32	52.2±10.2	5.58±0.08	2.43±0.31	73.3±13.7
WT	8	5.61±0.02	6.34±1.01	48.6±5.3	5.74±0.02	3.72±0.12	64.1±7.6
*P*		<0.001	<0.05	NS	<0.01	<0.001	NS

pCa_50_, -log[Ca^2+^] required for generation of 50% of maximal force. *n*, number of skinned fibers studied. Data: mean±SE. *P* values correspond to the comparison between *Tnnt2*^R141W/+^and WT fibers at the same sarcomere length. NS, not significant; WT, wildtype.

### Altered Ca^2+^ transients and action potentials in whole hearts

**Fig 7:**
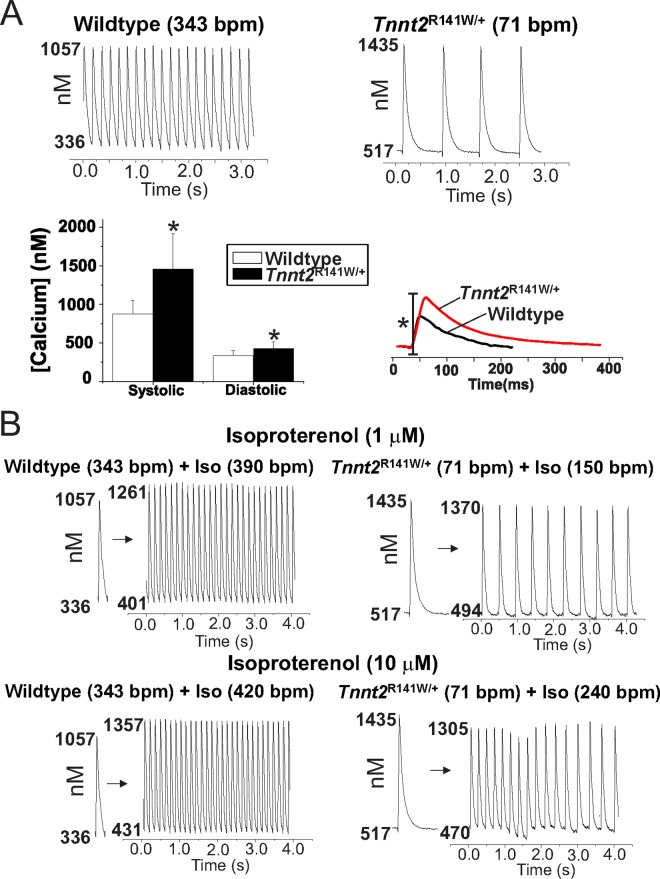
Representative Ca^2+^ transients from wildtype (WT) and *Tnnt2*^R141W/+^ hearts. **(A)** Langendorff perfused hearts were loaded with Rhod-2/AM and imaged on a 16x16 element photodiode array to map Ca^2+^ transients (CaT). Spontaneously beating, *Tnnt2*^R141W/+^ hearts exhibited a markedly slower intrinsic sinus rhythm heart rate (71±12 bpm, top right traces) compared to wildtype hearts (343±52 bpm top left traces; *n* = 6 each, *P*<0.03). Calibration of Rhod-2 through measurements of F_max_ (maximum Rhod-2 fluorescence when saturated with Ca^2+^) and F_min_ (minimum Rhod-2 fluorescence when all Ca^2+^ is washed out and chelated with EGTA) detected increases in diastolic and peak systolic cytosolic Ca^2+^ in *Tnnt2*^R141W/+^ compared to wildtype hearts (lower left panel). *Tnnt2*^R141W/+^ hearts had a longer rise time of [Ca^2+^]_i_, (WT = 14.3±2.09 ms, *n* = 4 and *Tnnt2*^R141W/+^ = 17.20±0.67, *n* = 6; *P*<0.05), and a longer time to recover to diastolic[Ca^2+^]_i_. Lower right traces show the superposition of normalized CaTs from a wildtype and a *Tnnt2*^R141W/+^ heart recorded from the center of the left ventricles, when both hearts were paced at the same cycle length (350 ms). The superposition of CaTs exposes marked differences in Ca^2+^ dynamics associated with Ca^2+^ desensitization in *Tnnt2*^R141W/+^ hearts. **(B)** *Tnnt2*^R141W/+^ hearts exhibited lower intrinsic heart rates and lower peak heart rates in response to isoproterenol at 1 and 10 μM concentrations, compared to wildtype, *P*<0.001.

**Fig 8:**
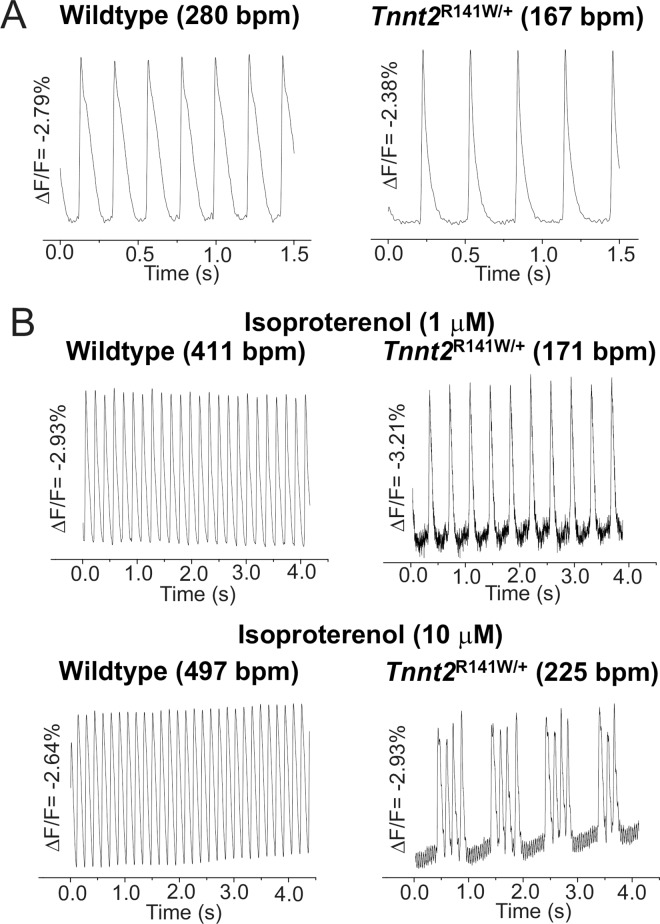
Representative APs recorded from wildtype and *Tnnt2*^R141W/+^ hearts. **(A)** Spontaneously beating *Tnnt2*^R141W/+^ hearts had an intrinsically lower heart rate. **(B)** *Tnnt2*^R141W/+^ hearts exhibited relatively small increases in heart rate at 1 and 10 μM concentrations.

**Fig 9:**
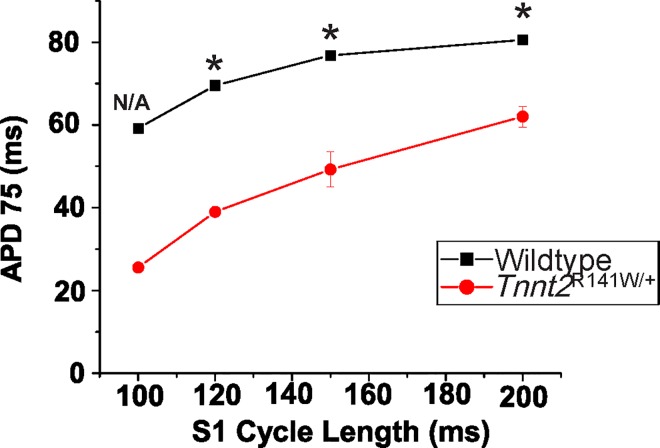
Pacing kinetics. AP durations were shorter in *Tnnt2*^R141W/+^ relative to wildtype hearts at all pacing cycle lengths. *n* = 4 for WT, *n* = 3 for all *Tnnt2*^R141W/+^ because not all *Tnnt2*^R141W/+^ hearts could be paced at faster rates or shorter cycle lengths. *, *P*<0.001 vs. wildtype.

To investigate further the impact of the *Tnnt2* R141W mutation on Ca^2+^ homeostasis, we performed optical mapping studies on isolated 8-week-old *Tnnt2*^R141W/+^ hearts and wildtype controls using the Ca^2+^ indicator Rhod-2/AM (Fig 7). Studies indicated 54% and 20% increases, respectively, in peak systolic and end diastolic [Ca^2+^]_i_ in *Tnnt2*^R141W/+^ relative to wildtype hearts. We also observed a prolonged systolic rise and diastolic fall in [Ca^2+^] in *Tnnt2*^R141W/+^ relative to wildtype hearts. *Tnnt2*^R141W/+^ hearts exhibited a slower intrinsic sinus rhythm heart rate compared to wildtype hearts and exhibited lower peak heart rates in response to β-adrenergic stimulation with isoproterenol; moreover, exposure to higher concentrations of isoproterenol led to sustained dysrhythmias (Figs 7 and 8).Optical mapping of APs with the voltage-sensitive indicator di-4-ANEPPS showed that AP durations were shorter in *Tnnt2*^R141W/+^ hearts compared to wildtype hearts (Figs 8 and 9) and this property of longer Ca^2+^ transients but shorter AP durations would produce a substrate highly prone to arrhythmias.

### Altered expression of Ca^2+^ handling proteins

**Fig 10:**
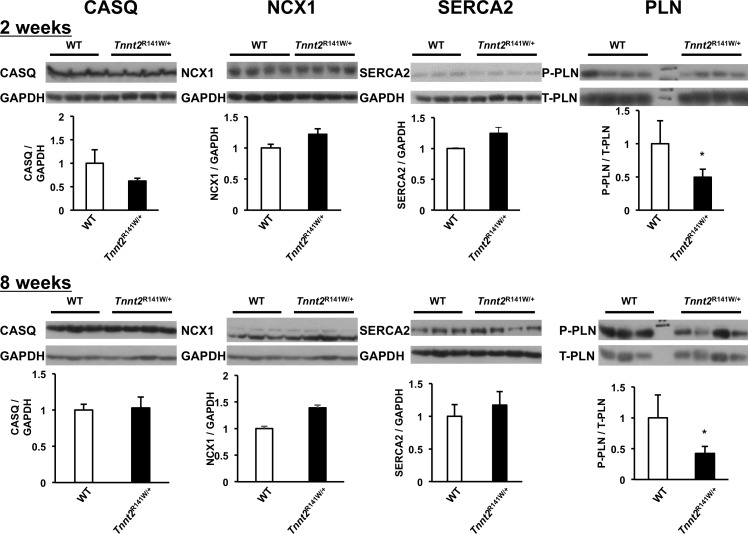
Expression of Ca^2+^ handling proteins. Immunoblots and densitometry analysis of total cardiac protein extracts from wildtype (WT) and *Tnnt2*^R141W/+^hearts at ages 2 and 8 weeks. CASQ2, calsequestrin 2; NCX1, sodium/calcium exchanger; P-PLN, Ser^16^ phosphorylated phospholamban; SERCA2, sarcoplasmic reticulum cardiac Ca^2+^ ATPase 2; T-PLN, total phospholamban. *, *P*<0.01 versus wildtype.

The molecular basis for increased Ca^2+^ transients in conjunction with Ca^2+^ desensitization is uncertain. We hypothesized that it is associated with perturbations in Ca^2+^ handling proteins. By immunoblot, we quantified several proteins mediating Ca^2+^ storage and release from the sarcoplasmic reticulum and Ca^2+^ extrusion from the cell in *Tnnt2*^R141W/+^ hearts at ages 2 and 8 weeks, namely calsequestrin 2 (CASQ2), the sodium/calcium exchanger (NCX1), sarcoplasmic reticulum cardiac Ca^2+^ ATPase 2 (SERCA2), and phospholamban (PLN) (Fig 10). At ages 2 and 8 weeks, protein expression of CASQ2, NCX1, and SERCA2 were similar among wildtype and *Tnnt2*^R141W/+^ hearts. The relative expression of phosphorylated PLN to total PLN was significantly decreased in *Tnnt2*^R141W/+^ hearts relative to wildtype hearts at both 2 and 8 weeks of age.

### No change in DCM phenotype by crossing *Tnnt2*^R141W/+^ mice with *Pln*^-/-^ mice

Phospholamban knockout mice (*Pln*^-/-^), constructed by Kranias and colleagues [17], exhibit hypercontractility associated with increased uptake, sequestration, and systolic release of Ca^2+^ from the sarcoplasmic reticulum. On echocardiography, *Tnnt2*^R141W/+^/*Pln*^-/-^ mice exhibited little or no improvement in LVEDD or FS (Table 4) relative to *Tnnt2*^R141W/+^.

## Discussion

In this study, we used homologous recombination to replace the endogenous *Tnnt2* murine gene with an allele carrying the R141W mutation previously identified in a human family with DCM [14]. Mice heterozygous for the mutation (*Tnnt2*^R141W/+^) recapitulated the human phenotype, developing left ventricular dilation and reduced contractility. We observed a gene dosage effect with attenuation of the phenotype in *Tnnt2*^R141W/+^ mice by transgenic overexpression of wildtype *Tnnt2* transcript. We also documented poorer survival among male *Tnnt2*^R141W/+^ mice relative to females. Similar to prior *in vitro* investigations of the R141W mutation [8–11], our *ex vivo* biomechanical studies suggest that Ca^2+^ desensitization is a primary mechanism for the development of DCM. To clarify further the physiologic consequences of the R141W mutation, we conducted optical mapping studies. We found that isolated *Tnnt2*^R141W/+^ hearts had increased peak systolic and end diastolic intracellular Ca^2+^ ([Ca^2+^]_i_), prolonged systolic rise and diastolic fall in [Ca^2+^]_i_, shortened action potential durations, and a lower peak heart rate with adrenergic stimulation. Furthermore, analysis of Ca^2+^ handling proteins shows a reduction in phospholamban phosphorylation. Crossing *Tnnt2*^R141W/+^ mice with phospholamban knockout (*Pln*^-/-^) mice [17], that exhibit hypercontractility and increased Ca^2+^ uptake, sequestration, and release from the sarcoplasmic reticulum, did not attenuate the DCM phenotype. Consistent with prior *in vitro* investigations of the R141W mutation [8–11], as well as *in vitro* and *in vivo* investigations of other *TNNT2* DCM mutations, our data suggest that reduced myofilament Ca^2+^ binding affinity caused by the R141W mutation leads to an adaption of the system by increasing the amplitude of Ca^2+^ transients. We speculate, however, that the higher Ca^2+^ transients reduce the range of physiological conditions in which the muscle can operate. It cannot contract at normal maximum heart rates in the presence of physiological beta-adrenergic agonists like isoproterenol, and is predisposed to arrhythmias.

In our current study, we found that the transgenic overexpression of wildtype *Tnnt2* transcript, superimposed on mutant R141W *Tnnt2* transcript, in *Tnnt2*^R141W/+^/TG^WT^ mice largely abrogated the DCM phenotype observed in *Tnnt2*^R141W/+^ mice. We speculate that this attenuation occurs because only a very small proportion of mutant cTnT protein is still expressed and present in the sarcomeres of *Tnnt2*^R141W/+^/TG^WT^ mice. However, it should be noted that we were able to measure only relative expression of wildtype and mutant *Tnnt2* transcripts in these mice, which may not completely reflect the relative expression of wildtype and mutant cTnT protein. The evident gene dosage effect in which phenotype severity is correlated with relative expression of mutant transcript is consistent with our previous findings in *Tnnt2* null mice and transgenic mice with the *Tnnt2* K210Δ mutation suggesting that *TNNT2* mutations lead to DCM by production of abnormally functioning protein rather than haploinsufficiency, and that greater expression of mutant protein leads to a more severe phenotype [4].

The mechanism of the difference in survival between male and female mice is uncertain. However, this difference parallels that seen in other murine models of cardiomyopathy, and is thought to be a reflection of divergent hormonal effects on multiple aspects of cardiac biology [32]. *Tnnt2*^R141W/+^ male mice and *Tnnt2*^R141W/+^ female mice exhibited equally poor fractional shortening (FS) (Table 3). Therefore, we speculate that the increased mortality evident in *Tnnt2*^R141W/+^ male mice may be secondary to an increased arrhythmic burden rather than pump failure. However, sex differences in arrhythmic burden remain to be documented.

Although *Tnnt2*^R141W/+^ mice exhibit early left ventricular dilation and dysfunction at age 6 weeks, it appears that there is no progression of dilation and dysfunction through ages 12 and 30 weeks. Although it is reasonable to infer that ventricular remodeling should progress with age, it should be noted that in the study reporting human subjects with the *TNNT2* R141W mutation [14], LVEF was not completely correlated with age. There may be inherent differences in biology between mice and humans, such that adaptive mechanisms in the mouse heart protect it from progressive ventricular remodeling as it ages. Finally, our study may be suffering from survivorship bias, so that mice only mice who exhibit less ventricular remodeling or arrhythmic burden survive to undergo echocardiography at later ages.

Although many of the molecular mechanisms of ventricular remodeling in heart failure have been elucidated, few investigations have been performed to determine which of these mechanisms are active in hereditable DCM secondary to single gene mutations. There is a possible activation of the cardiotrophin-1 hypertrophic pathway manifested by a statistically significant increase in Tyr^1007/1008^ phosphorylation of Jak-2 (and a trend towards increased expression of cardiotrophin-1 that did not meet statistical significance). This pathway has recently been shown to increase I_CaL_, Ca^2+^ sparks, spontaneous Ca^2+^ waves, and afterdepolarizations [33]. Therefore, activation of the cardiotrophin-1 pathway may be related to the hypertrophy, the increased intracellular Ca^2+^, and the arrhythmic predisposition observed in these mice. Several signaling pathways associated with ventricular remodeling appear not to be active in *Tnnt2*^R141W/+^ hearts such as the ratio of active, Ser^473^ phosphorylated form of Akt (pAkt) to total Akt. Akt appears to mediate physiological growth of the heart [34] and physiological hypertrophy in response to exercise [35] and appears to mitigate against decompensation following prolonged pressure overload. The phosphorylated, active form of p38 MAPK was downregulated, and the phosphorylated, active forms of ERK1 and ERK2 MAPKs were unchanged. The MAPK pathways may similarly mediate adaptive remodeling in the heart [36]. Therefore, a lack of activation or actual impairment of some adaptive remodeling pathways may be mechanistically related to the pathological remodeling that characterizes DCM. However, some adaptive pathways are activated. We have demonstrated increased expression of calcineurin, which appears to play a protective role in DCM, likely through an anti-apoptotic pathway [37].

Consistent with prior studies, we have identified Ca^2+^ desensitization as a primary mechanism for impaired cardiac contractility in *Tnnt2*^R141W/+^ mice. Biomechanical studies of skinned fibers from *Tnnt2*^R141W/+^ hearts indicated reduced myofilament Ca^2+^ sensitivity, without any changes in maximally activated force or length-dependent changes in Ca^2+^ sensitivity. This Ca^2+^ desensitization appears not to be attributable to TnI phosphorylation. Recently, *in vitro* motility assays have shown that DCM-causing mutations in thin filament proteins, including the *TNNT2* R141W mutation, abolish the relationship between myofilament Ca^2+^ sensitivity and TnI phosphorylation by PKA [9]. Although the maximally activated force generation was not impaired, it is likely that at physiological intracellular Ca^2+^ concentrations the observed Ca^2+^ desensitization leads to decreased force generation *in vivo*. Ca^2+^ desensitization has been observed in *in vitro* studies of R141W mutant cTnT protein [8–11], and similar observations have been made for other DCM mutations [38]. We [4] and others [7] have documented Ca^2+^ desensitization in *in vivo* mouse models of the *Tnnt2* K210Δ DCM mutation. Similar to our current findings, these mice exhibited unaltered maximally activated force generation.

To understand the physiologic consequences of the R141W mutation, we performed optical mapping studies in *Tnnt2*^R141W/+^ mice that showed increased diastolic intracellular Ca^2+^ ([Ca^2+^]_i_), a prolonged systolic rise of [Ca^2+^]_i_, increased peak systolic [Ca^2+^]_i_, and a prolonged diastolic fall in [Ca^2+^]_i_. These findings suggest increased Ca^2+^ bioavailability to the sarcomere to compensate for decreased Ca^2+^ sensitivity. Furthermore, the increased Ca^2+^ bioavailability is associated with a reduction in phosphorylated phospholamban expression. The prolonged Ca^2+^ transients may limit the ability of *Tnnt2*^R141W/+^ hearts to increase heart rates and to respond to adrenergic stimulation. In fact, they are predisposed to arrhythmias when exposed to adrenergic stimulation. The reduction in phosphorylated phospholamban suggests greater inhibition of SERCA2, which would lead to the observed prolongation in diastolic fall in [Ca^2+^]_i_ and the increased end diastolic [Ca^2+^]_i_. These observations may also be related to the decreased myofilament affinity for Ca^2+^, so that there is more free, unbound Ca^2+^. However, there are likely also other complex mechanisms at play that remain to be identified, because decreased phosphorylation of phospholamban is generally associated with decreased Ca^2+^ transient amplitudes rather than our observed increased Ca^2+^ transient amplitudes. Our findings in *Tnnt2*^R141W/+^ hearts are similar to those in *Tnnt2*^K210Δ/+^ hearts, which exhibited increased peak amplitude of the Ca^2+^ transient in cardiomyocytes [7]. The abnormal phenotype in *Tnnt2* ^K210Δ/+^ hearts was attenuated by a positive inotropic agent, pimobendan, which directly increases myofilament Ca^2+^ sensitivity [7]. In contrast, however, genetic ablation of cardiac *Pln*, which increases the uptake, sequestration, and systolic release of Ca^2+^ in the sarcoplasmic reticulum, had no salutary effect on the phenotype of *Tnnt2*^R141W/+^ mice. We speculate that the increased Ca^2+^ bioavailability associated with *Pln* ablation had no additional benefit in *Tnnt2*^R141W/+^ mice in which sarcoplasmic Ca^2+^ was already increased. The R141W mutation is located within the tropomyosin binding domain of cTnT, and has been shown to increase the affinity of cTnT for tropomyosin [8]. Thus, this mutation may lead to a gain of function, decreasing Ca^2+^ sensitivity of the troponin complex.

Decreases in adrenergic sensitivity are generally expected to increase Ca^2+^ sensitivity. β-adrenergic stimulation leads to a PKA phosphorylation-dependent decrease in myofilament Ca^2+^ sensitivity, an associated increase in the rate of Ca^2+^ dissociation from TnC, and an increase in the rate of relaxation that facilitates more rapid heart rates and increased contractility. However, Marston and colleagues have documented uncoupling of Ca^2+^ sensitivity from phosphorylation status in several DCM mutations *in vitro* [39], and a concordant decrease in β-adrenergic sensitivity *in vivo* [40].

A C57BL/6J transgenic mouse model with cardiac overexpression of R141W mutant human cTnT has been previously reported [41]. To the extent that this transgenic model has been characterized, there is general concordance between its phenotype and that of the knock-in model reported here. In the transgenic mice, it was estimated that mutant *Tnnt2* transcript was expressed at a level 1.5–2.0-fold greater than endogenous wildtype *Tnnt2* transcript, a proportion that likely was greater than the approximate parity between mutant and wildtype *Tnnt2* transcript in *Tnnt2*^R141W/+^ mice. (However, the relative expression of wildtype and mutant cTnT protein may not be exactly the same as the relative expression of wildtype and mutant *Tnnt2* mRNA transcript.) Transgenic mice exhibited similar LV dilation and impaired contractility, and exhibited some of the same changes in signaling pathways as *Tnnt2*^R141W/+^ mice. In contrast to *Tnnt2*^R141W/+^ mice, however, the transgenic mice exhibited a greater degree of interstitial fibrosis. Another transgenic mouse model with cardiac overexpression of R141W mutant human cTnT [42] also exhibited decreased Ca^2+^-sensitivity of myofibrillar protein ATPase activity.

This report is one of only a few to describe a gene-targeted knock-in murine models of human mutations in *TNNT2* leading to DCM, and to describe the consequences of the mutation on gene dosage, sex-dependent survival, molecular remodeling pathways, electrophysiological function, and Ca^2+^ sensitivity, transients, and homeostasis. Our data point to a broad and central role of Ca^2+^ in the pathophysiology of DCM, with impaired Ca^2+^ sensitivity as possibly the initial direct consequence of the *TNNT2* mutation. The decreased sensitivity leads to perturbations in Ca^2+^ transients and arrhythmogenesis. Combined with other studies, our findings are consistent with the paradigm that intracellular Ca^2+^ homeostasis is a major player in the pathogenesis of both DCM and HCM, with the distinct phenotypes resulting from divergent effects of genetic mutations on Ca^2+^ sensitivity.
